# Genome Sequence of a Spontaneous Nonhemolytic Mutant of *Mannheimia haemolytica* 16041065 GH

**DOI:** 10.1128/genomeA.01720-16

**Published:** 2017-04-06

**Authors:** Sahlu Ayalew, Anthony W. Confer, Richard D. Hansen, Matthew Brian Couger

**Affiliations:** a Department of Veterinary Pathobiology, Center for Veterinary Health Sciences, Oklahoma State University, Stillwater, Oklahoma, USA; b SolidTech Animal Health Inc., Newcastle, Oklahoma, USA; c Oklahoma State University High Performance Computing Center, Stillwater, Oklahoma, USA

## Abstract

We report here the draft genome sequence of a spontaneous nonhemolytic mutant of *Mannheimia haemolytica* 16041065 GH. This mutant arose during routine passage and was devoid of hemolytic activity on standard blood agars. This genome sequence had a total size of 2.7 Mb with an *N*_50_ of 117 kb.

## GENOME ANNOUNCEMENT

Several bacteria, including *Mannheimia haemolytica* and numerous viruses, contribute to what is commonly referred to as bovine respiratory disease complex. However, the bacterial pathogen that is most commonly associated with the acute, severe form of the disease, and that is readily isolated from lung lesions, is *M. haemolytica* (1). *Mannheimia* (formerly *Pasteurella*) *haemolytica* is a Gram-negative coccobacillus that is readily isolated from nasal swabs of domestic ruminants such as cattle, sheep, and goats, where it constitutes the normal flora of the upper respiratory tract (2). Numerous serotypes of this bacterium have been isolated and characterized (3, 4). This otherwise innocuous opportunistic pathogen aggressively replicates when the host’s defense mechanisms are compromised by any number of stressful factors (5). Under these conditions, *M. haemolytica* can be inhaled into the lower respiratory tract and cause severe, and often fatal, pneumonia (1). Studies have shown that several characteristics or activities of this bacterium contribute to its virulence. An exotoxin, leukotoxin, is considered the most important virulence factor associated with *M. haemolytica* (6). Leukotoxin is responsible for *M. haemolytica* hemolysis on blood agar and causes leukocyte necrosis and apoptosis *in vivo*, thus allowing the bacterium to escape phagocytosis and killing by neutrophils and macrophages (7, 8).

One of the cultural characteristics of *M. haemolytica* on brain heart infusion (BHI) blood agar plates is its ability to cause clearing around the colonies, a feature also known as β-hemolysis (8). During routine transfers in our laboratory of an otherwise β-hemolytic strain, a spontaneous γ-hemolytic (nonhemolytic) mutant was observed. The identity of this γ-hemolytic mutant was confirmed as *M. haemolytica* serotype 1 by matrix-assisted laser desorption ionization–time of flight mass spectrometry and serology (9, 10).

Genomic DNA was extracted from a well-isolated colony grown in BHI broth using a Wizard Genomic DNA purification kit (Promega, Madison, WI, USA). The purity of the genomic DNA data was tested by measuring absorbance at 260 and 280 nm (ratio of 260/280). The integrity of the data was further confirmed by resolving uncut and *EcoR*I digested forms on 1% agarose gel.

All sequence reads for *M. haemolytica* 16041065 GH were quality-filtered with standard Illumina filtering settings, resulting in 250 Mb of quality sequence data. Quality-filtered reads were assembled to produce the genome using the short-read de Brujin graph assembly (11) program Velvet (12). Run-time settings for Velvet were a *k*-mer value of 77 and a minimum contig coverage value of 7×. All gene models were created using the prokaryotic gene-calling software package Prodigal (13) on the Velvet genome assembly. The Velvet genome assembly had a total size of 2,675,629 bp, 68 scaffolds, a scaffold *N*_50_ of 117 kb, and 2,588 predicted proteins. All called gene models were annotated using a combination of homology comparison, domain prediction, and annotation. Programs used for analysis of all gene models were NCBI BLAST C++ homology search (14) and HMMER version 3.0 hmmscan (15) against the Pfam version 26.0 database (16). All genes were annotated for functional significance using the UniProt database (17).

### Accession number(s).

This whole-genome shotgun project has been deposited in GenBank under the accession number MLYM00000000. The version described in this paper is the first version, MLYM01000000.
